# Micro-Nano Carbon Structures with Platelet, Glassy and Tube-Like Morphologies

**DOI:** 10.3390/nano9091242

**Published:** 2019-08-31

**Authors:** Mingqiang Liu, Juntong Huang, Qingming Xiong, Suqing Wang, Zhi Chen, Xibao Li, Qianwei Liu, Shaowei Zhang

**Affiliations:** 1 The School of Materials Science and Engineering, Nanchang Hangkong University, Nanchang 300063, China; 2 College of Engineering, Mathematics and Physical Sciences, University of Exeter, Exeter EX4 4QF, UK

**Keywords:** nanoplatelets exfoliated from graphite, three-roll milling, catalytic growth of CNTs, phenol-formaldehyde resin, multidimensional nanocarbon structures

## Abstract

Carbon source precursors for high-grade, clean, and low-carbon refractories were obtained by in situ exfoliation of flake graphite (FG) and phenol–formaldehyde resin (PF) composites with three-roll milling (TRM) for the fabrication of graphite nanoplatelets. In addition, by using Ni(NO_3_)_2_·6H_2_O as a catalyst in the pyrolysis process, multidimensional carbon nanostructures were obtained with coexisting graphite nanoplatelets (GNPs), glassy carbon (GC), and carbon nanotubes (CNTs). The resulting GNPs (exfoliated 16 times) had sizes of 10–30 μm, thicknesses of 30–50 nm, and could be uniformly dispersed in GC from the PF pyrolysis. Moreover, Ni(NO_3_)_2_·6H_2_O played a key role in the formation and growth of CNTs from a catalytic pyrolysis of partial PF with the V–S/tip growth mechanisms. The resulting multidimensional carbon nanostructures with GNPs/GC/CNTs are attributed to the shear force of the TRM process, pyrolysis, and catalytic action of nitrates. This method reduced the production costs of carbon source precursors for low-carbon refractories, and the precursors exhibited excellent performances when fabricated on large scales.

## 1. Introduction

Carbon-containing refractories, which exhibit excellent thermal shock resistances and corrosion resistances because of the additional carbon, have been widely applied over the last few decades [1,2]. However, the high carbon contents (10–20 wt.%) in current commercialized carbon-containing refractories leads to serious complications, such as inferior oxidation resistance, high thermal conductivity, high carbon pick-up in molten steel, and corresponding degradation of the mechanical properties of the products [3,4,5]. Therefore, the carbon contents of refractories must be reduced to appropriate levels (<10 wt.%) [6]. The increasing demand for high-quality steelmaking in the metallurgical industry and the rapid development of external refining technologies have brought more attention to low-carbon (<10 wt.%) and ultra-low-carbon (<5 wt.%, ideally <3 wt.%) refractories.

To reduce the carbon contents in refractories, researchers have investigated a series of carbon sources to replace the traditional one. Tradition flake graphite (FG) (a major carbon source for carbon-containing refractories) does not meet the requirements of advanced steelmaking technology and low-carbon carbon-containing refractories (LCCRs). Moreover, researchers have devoted a lot of effort to developing LCCRs by adding nano-sized carbon sources [7,8,9]. First, carbon black (CB), as a zero-dimensional nano-sized carbon source, replaced FG partially or entirely. Thereby, it reduced the carbon content and improved the mechanical performance and thermal shock resistance of Al_2_O_3_–C LCCRs [10,11]. To enhance the strength and thermal shock resistance further, carbon nanotubes (CNTs) and carbon fibers (CFs) were employed as one-dimensional carbon sources in the LCCRs because of their excellent mechanical properties, such as high tensile strengths, elastic moduli, and bending strengths [12,13,14,15,16,17]. Recently, graphene, graphite oxide nanosheets (GONs), or graphite nanoplatelets (GNPs) were added as two-dimensional carbon sources to Al_2_O_3_–C LCCRs, which strengthened their cold moduli of rupture, flexural moduli, and force and displacement curves [18,19,20,21]. However, the previously mentioned nano-carbon sources (CB, CNTs/CFs, graphene, and GONs) are expensive because of their complicated fabrication processes, and it becomes very difficult to disperse/distribute them homogeneously in the whole LCCR matrix. This restrains their large-scale applications in LCCRs and the potential for long-term development of high-quality steelmaking and external refining technologies. Therefore, a low-cost route for the large-scale preparation of an applicable carbon source precursor for LCCRs must be developed to meet the performance requirements of advanced steelmaking.

Commercialized carbon-containing refractories usually contain flake graphite (FG) as the carbon source and phenol–formaldehyde resin (PF) as the binder. Therefore, the commercial three-roll milling (TRM) method is proposed for the economic exfoliation of FG in PF. The results are graphite nanoplatelets (GNPs; micro-sized in transverse, and nano-sized in longitudinal directions) as the carbon source precursors for LCCRs [22,23]. To improve the strength and thermal shock resistance of LCCRs, we employed a catalytic approach for the partial conversion of PF into CNTs (in situ growth) and glassy carbon (GC) with nickel nitrate at 1000 °C. Thereby, micro/nano carbon structures with platelet, glassy, and tube-like morphologies (GNPs/GC/CNTs) were produced on large scales and could be used as inexpensive carbon precursors in LCCRs.

## 2. Experiments

### 2.1. Exfoliation of FG into GNPs

**Figure 1:**
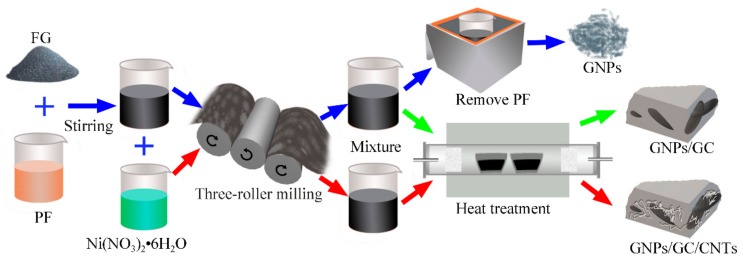
Preparation protocols for exfoliated Flake graphite (FG) samples (blue arrow), graphite nanoplatelets/glassy carbon (GNPs/GC) composites (green arrow), and multidimensional nanocarbon structure with GNPs/GC/carbon nanotubes (CNTs) (red arrow).

Flake graphite (FG) (approximately 150 μm; Qingdao Jintao Graphite Co., Ltd., Qingdao, Shandong, China) and PF resin (thermosetting PF resin 900A1; Hebei Zetian Chemical Co., Ltd., Hengshui, Hebei, China) were used as raw materials. As shown in Figure 1 (blue arrow), the investigated weight ratios between FG and PF were 1, 3, 5, 8, 12, and 15 wt.%. The mixture was heated in a water bath at 35 °C and stirred with an electric mixer (JJ-1) for 1 h. This way, the FG was uniformly dispersed in PF. Before the exfoliation process, the dispersed FG/PF mixture was cooled down to room temperature to restore the initial PF viscosity. Subsequently, a commercial TRM (Sichuan Changxinyuan Machinery Equipment Co., Ltd., Chengdu, Sichuan, China) was employed to disperse and exfoliate in situ the FG in the pure PF. The gaps between the rolls were set to 1:3:6. The FG/PF mixture was passed 1–16 consecutive times through the TRM at a constant speed. Furthermore, ethanol (AR) was added to the mixture, followed by ultrasonic cleaning and high-speed centrifugation to remove the PF. The resulting exfoliated product was dried in an oven at 80 °C for 12 h.

### 2.2. Pyrolysis of GNPs/PF Mixture

The pyrolysis of the obtained GNPs/PF mixtures with different FG contents after 16 exfoliations was conducted to prepare GNPs/GC composites in two steps. Initially, the GNPs/PF mixtures were directly solidified by firing the samples from ambient temperature to 80 °C at a rate of 3 °C min^−1^ and holding the temperature for 1 h. Second, the temperature was increased to 120 °C at a rate of 3 °C min^−1^ and held for 1.5 h. Third, the temperature was increased to 180 °C at a rate of 2 °C min^−1^ and held for 3 h. Subsequently, the solidified composites were carbonized in an argon atmosphere at 1000 °C for 3 h (Figure 1; green arrow). Finally, the fired samples were furnace-cooled to room temperature.

### 2.3. Preparation of Multidimensional Carbon Structures with GNPs/GC/CNTs

Multidimensional carbon structures with GNPs/GC/CNTs were prepared with a similar procedure to that for the previously mentioned GNPs/GC composites. The only difference was the addition and dispersion of nickel nitrate hexahydrate (Ni(NO_3_)_2_·6H_2_O) into the raw materials before exfoliation (red arrow in Figure 1).

### 2.4. Characterization

To investigate the crystal structure of the exfoliated FG samples, X-ray diffraction (XRD; D8 Advance, Bruker, Karlsruhe, Germany) was employed. The morphologies of the different products were characterized with a scanning electron microscope (SEM; Nova NanoSEM 450, FEI, Hillsboro, OR, USA; 15 kV), a transmission electron microscope (TEM; Talos F200X, FEI, Hillsboro, Oregon, USA; 200 kV), selected-area electron diffraction (SAED), and energy-dispersive spectroscopy (EDS). The average particle size distribution of the exfoliated products was detected with a laser diffraction particle size analyzer (LA-950, Horiba, Kyoto, Japan). The thermal stabilities and weight losses of the samples were detected by a thermogravimetric analysis (TGA; Diamond TG/TDA, Perkin Elmer, Waltham, MA, USA) from room temperature to 1000 °C at a ramp rate of 10 °C·min^−1^ in an argon atmosphere of 200 mL·min^−1^.

## 3. Results and Discussion

### 3.1. Effect of Carbon Contents and the Exfoliated Times on the Resultant GNPs

**Figure 2:**
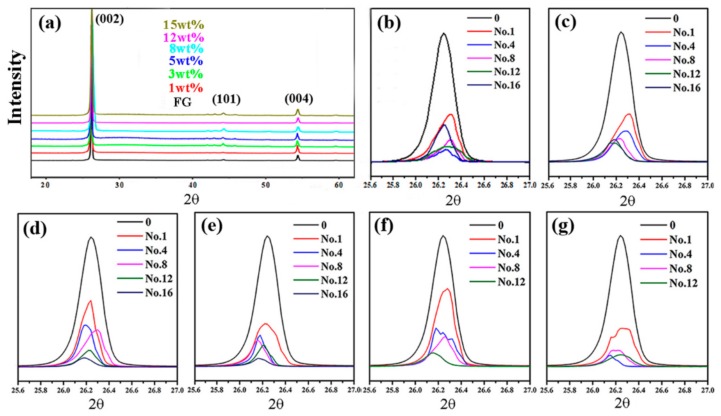
X-ray diffraction (XRD) pattern of products (**a**) with different graphite contents and 16 exfoliations; XRD patterns of products exfoliated at different times with different graphite contents: (**b**) 1 wt.%, (**c**) 3 wt.%, (**d**) 5 wt.%, (**e**) 8 wt.%, (**f**) 12 wt.%, and (**g**) 15 wt.%.

The products with different carbon contents exfoliated at different times with a TRM were characterized with XRD, as shown in Figure 2. Their XRD patterns showed no significant differences (Figure 2a) and were matched to original graphite. The intensities of the diffraction peaks corresponding to the (002) plane between 25.5° and 27° decreased almost continuously with increasing exfoliation time (Figure 2b–g). Thus, the crystallinity of the exfoliated FG was weakened. Moreover, the diffraction peaks corresponding to the (002) plane were shifted toward a lower two-theta angle (Figure 2b–g). According to the Bragg equation 2dsinθ = nλ, Scherrer equation τ = Kλ/βcosθ [24,25], and owing to the decreasing 2θ, the interplanar distance d_(002)_ should have been widened and the mean τ of the ordered domains corresponding to the (002) plane should decrease. Therefore, the products were thinned and refined after the exfoliation.

**Figure 3:**
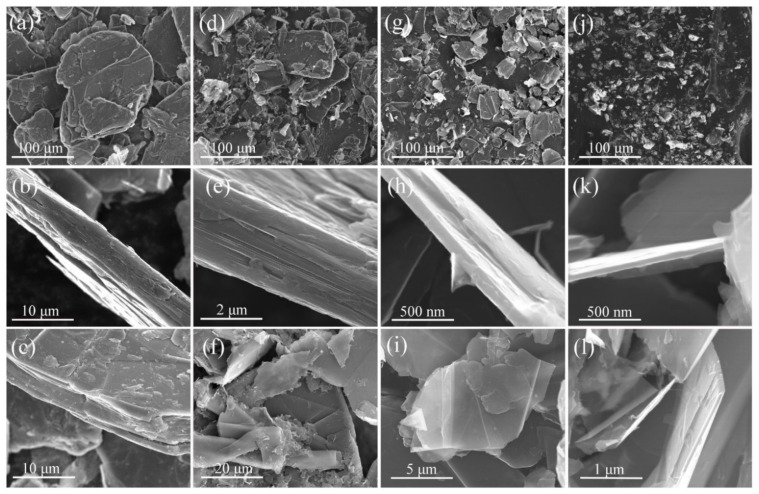
Size and morphology of (**a**–**c**) FG and products with 5 wt.% FG exfoliated at different times with a TRM (**d**–**f**): 1, (**g**–**i**): 8, and (**j**–**l**): 16 exfoliations.

To analyze the FG microstructure before and after the exfoliation, a SEM was employed. The size, thickness, and flexural structure of the original FG are provided in Figure 3a–c, respectively. The original FG had an average size of approximately 150 μm (Figure 3a), a thickness of approximately 7 μm (Figure 3b), and a platelet structure with thousands of layers (Figure 3c). Then, the SEM images of the exfoliated FG (1, 8, and 16 times) with 5 wt.% in PF (PF was removed before the SEM characterization) were studied to determine the changes in their structure/morphology. After the first exfoliation with the TRM, most particles decreased and exhibited average sizes of approximately 80 μm (Figure 3d–f) and thicknesses of 2–3 μm (Figure 3e). After eight exfoliations, the sizes and thicknesses of the products were approximately 45 μm and 100 nm (Figure 3g–i), respectively. After 16 exfoliations, the average particle size was only approximately 10 μm (Figure 3j), and the GNPs thicknesses were 10–30 nm (Figure 3h,l). Moreover, a transparent flexible film could be observed in Figure 3l. As previously mentioned, the sizes and thicknesses of the exfoliated FG decreased to those of micro/nano-sized graphite platelets owing to the TRM. However, the products maintained the typical platelet structure of graphite and exhibited better flexibility [26,27].

**Figure 4:**
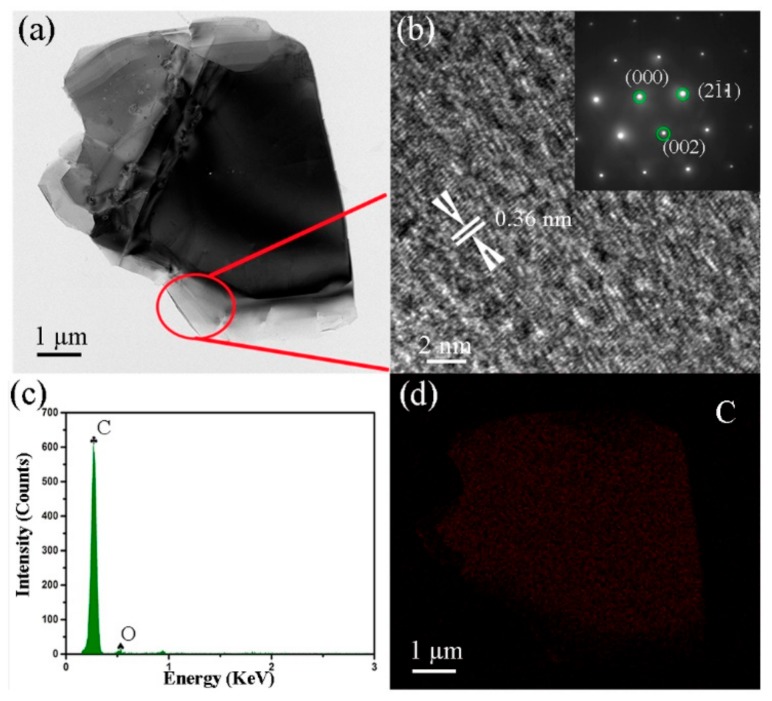
(**a**) Transmission electron microscope (TEM) image of exfoliated micro/nano-sized graphite sheets, (**b**) high resolution transmission electron microscope (HRTEM) image and diffraction pattern (inset), (**c**) energy spectrum of area, and (**d**) mapping of area.

Further details on the morphologies and crystal structures of the GNPs exfoliated 16 times from 5 wt.% FG are provided with TEM, high resolution transmission electron microscope (HRTEM), and SAED. A typical GNP is shown in Figure 4a. The edge area was thin because of the exfoliation effect. The marked lattice fringe d-spacing of 0.36 nm was in agreement with that of the (002) plane of the GNP (Figure 4b) and was larger than the 0.34 nm of the original FG [28]. The EDS and mapping results indicate that the GNPs maintained their carbon composition after 16 exfoliations with the TRM (Figure 4c,d). Moreover, the results reveal that the FG exfoliation in PF started at the FG edge and gradually proceeded to the center.

**Figure 5:**
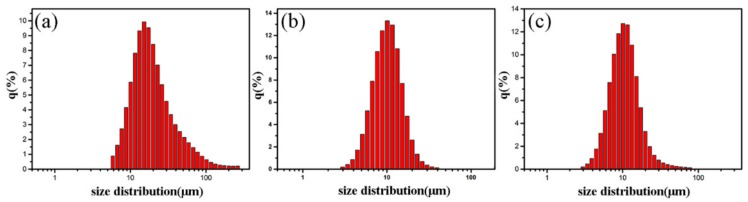
Particle size distributions of products with different contents of graphite exfoliated at different times: (**a**) 5 wt.% and 8 exfoliations, (**b**) 5 wt.% and 16 exfoliations, and (**c**) 8 wt.% and 16 exfoliations.

To determine the average particle size of the exfoliated samples, a laser diffraction particle size analyzer was used. Figure 5 shows the size distribution of the product after 8 and 16 exfoliations of graphite with 5 and 8 wt.% FG contents. The sizes of the exfoliated products with 5 wt.% FG decreased after 8–16 exfoliations. The minimal particle size decreased from 6 to 3 μm and the average size decreased from approximately 18.3 to 10.2 μm (Figure 5a,b). In addition, as the FG content was increased to 8 wt.%, the average size slightly increased and the large-particle size increased from 42 to 80 μm (Figure 5b,c). Thus, the FG content and exfoliation time both play important roles in the preparation of micro/nano graphite platelets with the TRM method.

### 3.2. Phase and Microstructure of GNPs/GC Composites

**Figure 6:**
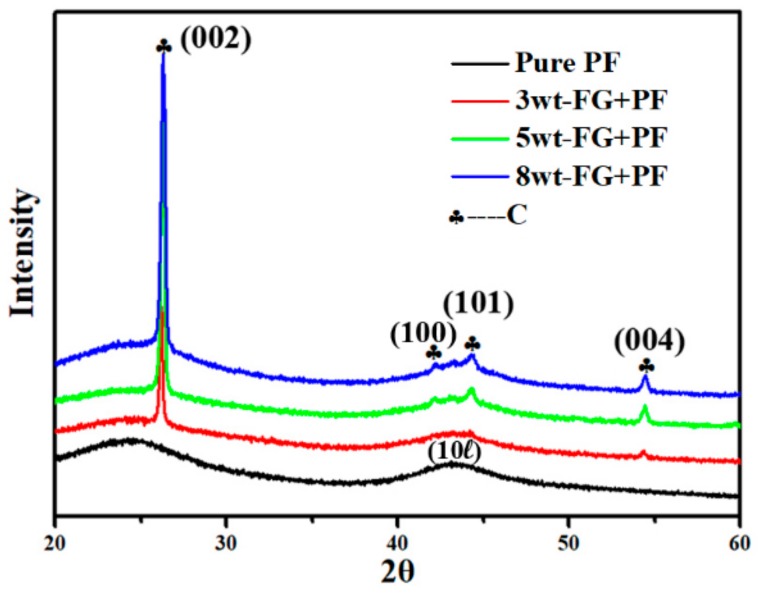
XRD patterns of products from pyrolysis of GNPs/phenol–formaldehyde (PF) mixture at 1000 °C with different FG contents exfoliated 16 times.

The XRD patterns of the products obtained from pure PF and different FG contents (3, 5, and 8 wt.%) in PF composites pyrolyzed at 1000 °C are shown in Figure 6. The pyrolyzed product without FG (i.e., pure PF) exhibited broad peaks at 24.5° and 43°, which corresponded to the (002) and (10ℓ) reflections of the turbostratic carbon structure, respectively. Thus, the PF transformed into GC. Moreover, the pyrolyzed product with FG displayed one more peak at 54.4°, which corresponded to the (004) reflection of the graphitic structure. By increasing the FG content, the diffraction peak intensities corresponding to the (002) and (004) planes of the pyrolyzed product were enhanced. The (002) peaks of the pyrolyzed products had multiple (asymmetric) peaks and were the result of the mixture of FG and GC structures. Furthermore, the (10ℓ) peak was split into (100) and (101) peaks for higher graphite content.

**Figure 7:**
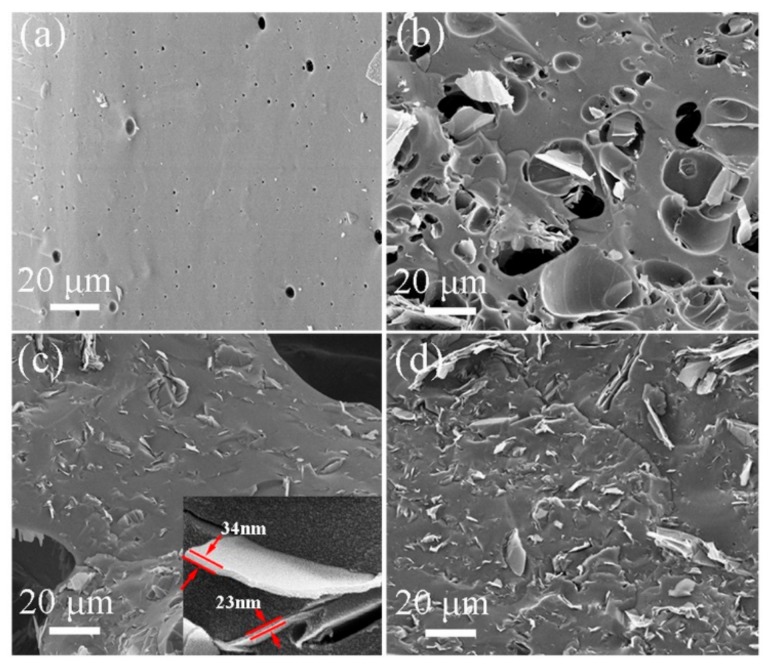
Scanning electron microscope (SEM) images of GNPs/PF composites pyrolyzed at 1000 °C with different FG contents exfoliated 16 times: (**a**) 0 wt.%, (**b**) 3 wt.%, (**c**) 5 wt.% (the inset shows the single exfoliated GNP), and (**d**) 8 wt.%.

The morphologies/microstructures of the products obtained from pure PF and different FG contents (3, 5, and 8 wt.%) in PF composites exfoliated 16 times with a TRM and pyrolyzed at 1000 °C were observed with a SEM. Figure 7a presents a relatively dense GC structure with some micro-scale holes in the product pyrolyzed from pure PF. The holes should have originated from gaseous phases like CO, CO_2_, CH_4_, and C_2_H_6_, and the H_2_O generated in the PF pyrolysis [29]. Owing to the addition of FG into PF, the holes became larger (Figure 7b,c). This was probably caused by the increasing FG viscosity after exfoliation and because GNPs were prevented from overflowing in the gaseous phases. The GNPs distribution in the fracture section of the product was more evident with an increasing FG content (Figure 7b–d). In particular, exfoliated GNPs with thicknesses of 20–40 nm were uniformly dispersed and closely embedded in the GC. This could be exploited to obtain stronger composites for future applications [30,31].

### 3.3. Phase and Microstructure of GNPs/GC/CNT Composites

**Figure 8:**
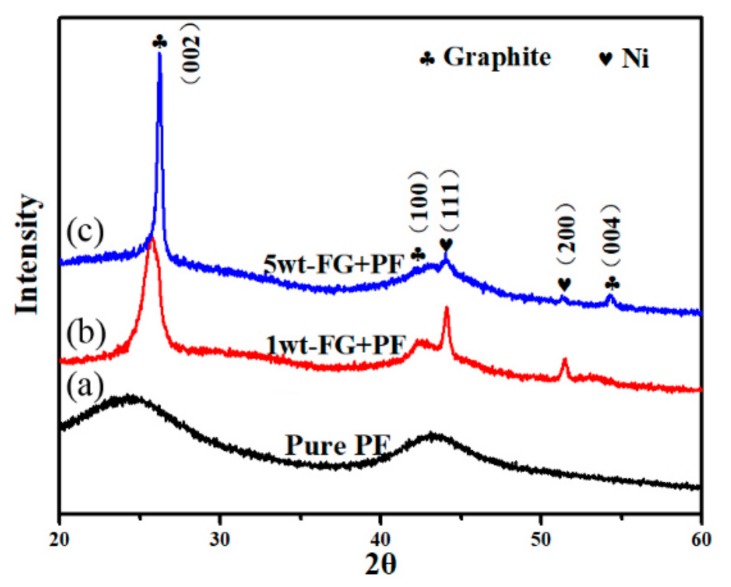
XRD patterns of different samples pyrolyzed at 1000 °C: (**a**) Pure PF, (**b**) 1 wt.% GNPs/PF with 1 wt.% Ni(NO_3_)_2_·6H_2_O, and (**c**) 5 wt.% GNPs/PF with 1 wt.% Ni(NO_3_)_2_·6H_2_O.

For the in situ growth of CNTs in GNPs/GC, we employed a catalytic approach for the partial conversion of PF into CNTs with nickel nitrate at 1000 °C. The XRD patterns of the products obtained from pure PF and FG/PF with 1 wt.% Ni(NO_3_)_2_·6H_2_O (exfoliated 16 times with TRM) are shown in Figure 8. The broad halos at 20°–30° (2θ) and 42°–47° indicate that the pyrolyzed carbon from the pure PF carbonized at 1000 °C was typically amorphous (Figure 8a). Similar results were observed for products obtained from FG/PF with 1 wt.% Ni(NO_3_)_2_·6H_2_O (1 or 5 wt.% FG), except for the detection of Ni catalysts and the graphite phase (i.e., GNPs) (Figure 8b,c). Nevertheless, the broad halos gradually decreased and became weaker with Ni catalysts, which suggested a gradual crystallization of amorphous carbon with the addition of Ni. The formation of Ni can be ascribed to the decomposition reaction of Ni(NO_3_)_2_·6H_2_O and the pyrolysis of PF at increasing temperatures.

**Figure 9:**
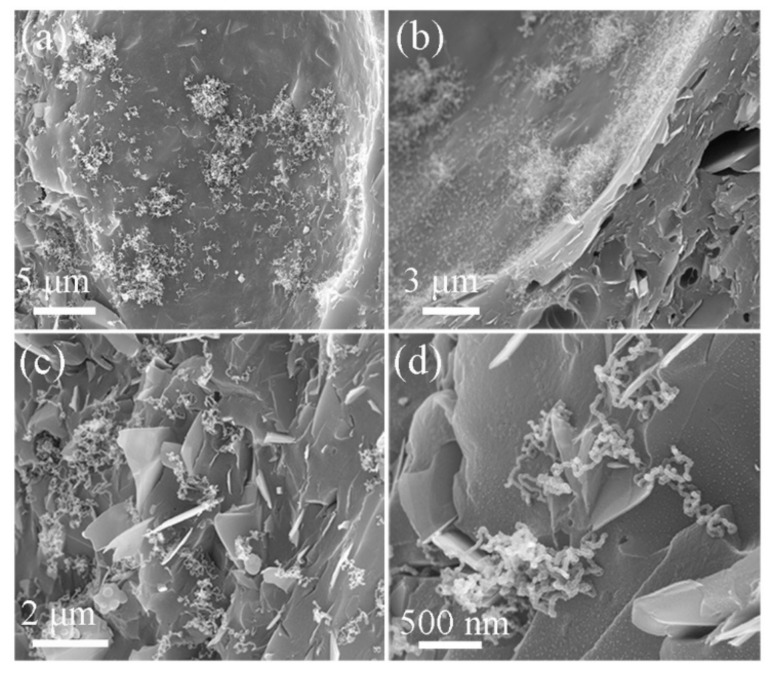
SEM images of samples with 5 wt.% GNP/PF with 1 wt.% Ni(NO_3_)_2_·6H_2_O pyrolyzed at 1000 °C in different regions. (**a**,**b**) Shows short-column carbon materials grew in bunches, (**c**,**d**) shows the exfoliated GNPs uniformly distributed in the curved, fibrous and nano-sized carbon materials.

To verify the effect of nickel nitrate on the product’s morphology, the SEM micrographs of the sample obtained from 5 wt.%-FG/PF with 1 wt.% Ni(NO_3_)_2_·6H_2_O (exfoliated 16 times with TRM and pyrolyzed at 1000 °C) are shown in Figure 9. Many short-column nano-sized carbon materials were deposited in the pores or at the surface of the pyrolyzed GC (Figure 9a,b). In addition, some of these grew in bunches and therefore exhibited a “clustered growth” mode. Moreover, many curved, fibrous, nano-sized carbon particles (approximately 1–2 μm in length and 20–30 nm in diameter) were inter-locked with each other and formed an intertexture on the pyrolyzed GC surface (Figure 9c,d). The exfoliated GNPs, uniformly distributed in the CNTs, were closely embedded into the GC. Thus, the product exhibited multidimensional micro/nano-sized carbon structures with platelet, glassy, and fibrous morphologies.

**Figure 10:**
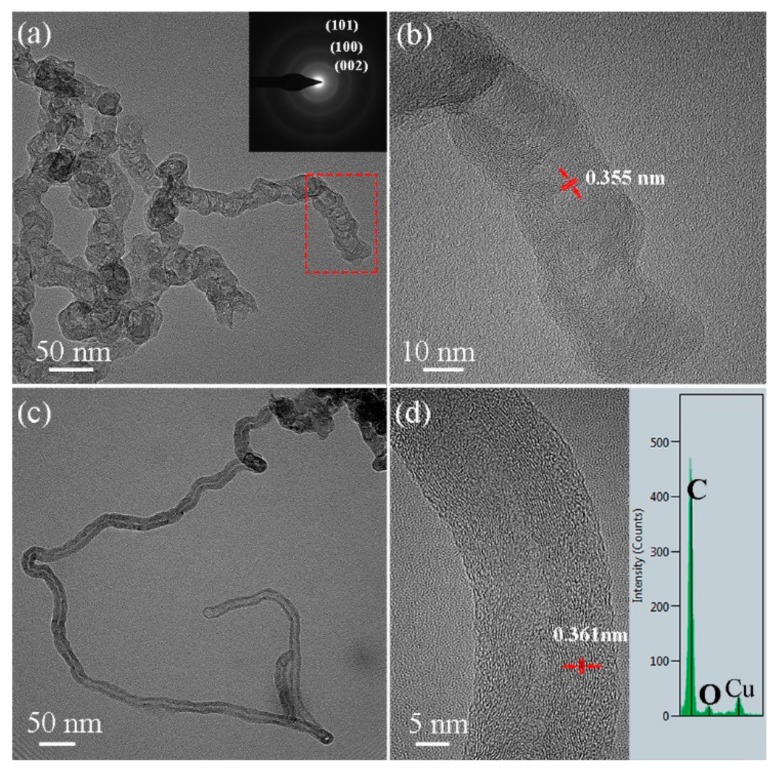
TEM and HRTEM images of samples with 5 wt.% GNPs/PF with 1 wt.% Ni(NO_3_)_2_·6H_2_O. (**a**,**b**) Shows the bamboo-like chains of carbon nanotubes (CNTs) (the inset in (**a**) shows the selected-area electron diffraction (SAED) pattern), (**c**,**d**) shows hollow CNTs (the inset in (**d**) shows the energy-dispersive spectroscopy (EDS) patterns).

Further details on the morphologies and crystal structures of the fibrous nano-sized carbon materials can be found in the TEM, HRTEM, SAED, and EDS results. According to Figure 10, the fibrous nano-sized carbon materials exhibited two kinds of tubular structures. Figure 10a,b presents bamboo-like chains of CNTs with diameters of 30–40 nm. The SAED image (inset in Figure 10a) of the CNTs includes three diffraction rings that correspond to the (002), (100), and (110) planes of graphite. Figure 10c,d presents hollow CNTs with diameters of 20–30 nm. Furthermore, according to the EDS pattern, the tubular structures were composed of carbon (inset in Figure 10d). The lattice spacings of 0.355 and 0.361 nm were larger than that of the (002) plane of graphite (0.335 nm) (Figure 10b,d). The expansion of the lattice fringe *d*-spacing reveals that the resultant CNTs had a low graphitization degree. Similar results were reported in References [32,33]. Thus, the product with Ni(NO_3_)_2_·6H_2_O formed multidimensional micro/nano-sized carbon structures with platelet, glassy, and tube-like morphologies. Such a multidimensional carbon structure could provide for an excellent performance in LCCR applications [34,35].

### 3.4. TG/DTA Results of PF, GNPs/PF, and GNPs/PF/Ni(NO_3_)_2_·6H_2_O

**Figure 11:**
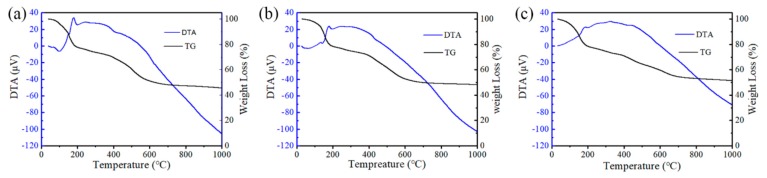
Thermogravimetric/differential thermal analysis (TG/DTA) curves of (**a**) pure PF, (**b**) 5 wt.% GNP/PF, and (**c**) 5 wt.% GNP/PF with 1 wt.% Ni(NO_3_)_2_·6H_2_O.

To thoroughly investigate the effects of the exfoliated GNPs and Ni(NO_3_)_2_·6H_2_O on the thermal stability and relative weight loss of PF, a thermogravimetric/differential thermal analysis (TG/DTA) was employed for the three samples. The results are shown in Figure 11. Based on the characteristics of the curves, the weight losses of the samples were 54.54% for pure PF (Figure 11a), 51.8% for 5 wt.% GNPs/PF (Figure 11b), and 48.6% for 5 wt.% GNPs/PF with 1 wt.% Ni(NO_3_)_2_·6H_2_O (Figure 11c). This implies ongoing chemical reactions (i.e., synthesis and/or oxidation between pyrolyzed PF and/or remnants of Ni(NO_3_)_2_·6H_2_O) or that the loss of compounds at the corresponding temperature came to an end [36]. Based on the trends of the curves in Figure 11a,b, the pyrolysis of pure PF and PF/FG was divided into three stages. The first stage exhibited a 3.6% mass loss at 25–100.3 °C, which should correspond to the overflow of water vapor in PF. At 100.3–700 °C, a significant weight loss occurred (approximately 45% in Figure 11a; 47.5% in Figure 11b). According to the DTA curves in Figure 8a,b, exothermic peaks appeared at 150 and 178 °C, which suggests that the ongoing chemical reactions (e.g., solidification and pyrolysis) led to a heat release. Moreover, a sharp, endothermic valley occurred between 178 and 241 °C, which should result from the starting release of gas (i.e., CO, CO_2_, CH_4_, H_2_, and C_2_H_6_) during the PF pyrolysis [29]. Above 700 °C, the weight loss rate was lower and only losses of 2.6 wt.% (pure PF) and 1.7 wt.% (PF/FG) occurred up to 1000 °C. This suggests that the exfoliated GNPs improved the residual carbon rate of PF. In contrast to Figure 11a,b, the exothermic peaks appeared at 183 and 324 °C for the sample with Ni(NO_3_)_2_·6H_2_O, which corresponded to the ongoing PF pyrolysis and decomposition of Ni(NO_3_)_2_·6H_2_O [37]. In addition, two unremarkable endothermic peaks occurred at 200 and 423 °C, which should have been due to the gas released in PF and the crystallization of metal Ni reduced from NiO, respectively [38]. Evidently, the addition of Ni(NO_3_)_2_·6H_2_O increased the residual carbon rate of PF. This was ascribed to the in situ formation of CNTs from the decomposed hydrocarbon in PF catalyzed by Ni.

### 3.5. In Situ Growth Mechanism of CNTs through Ni(NO_3_)_2_·6H_2_O–Catalytic Pyrolysis of GNP/PF Mixture

**Figure 12:**
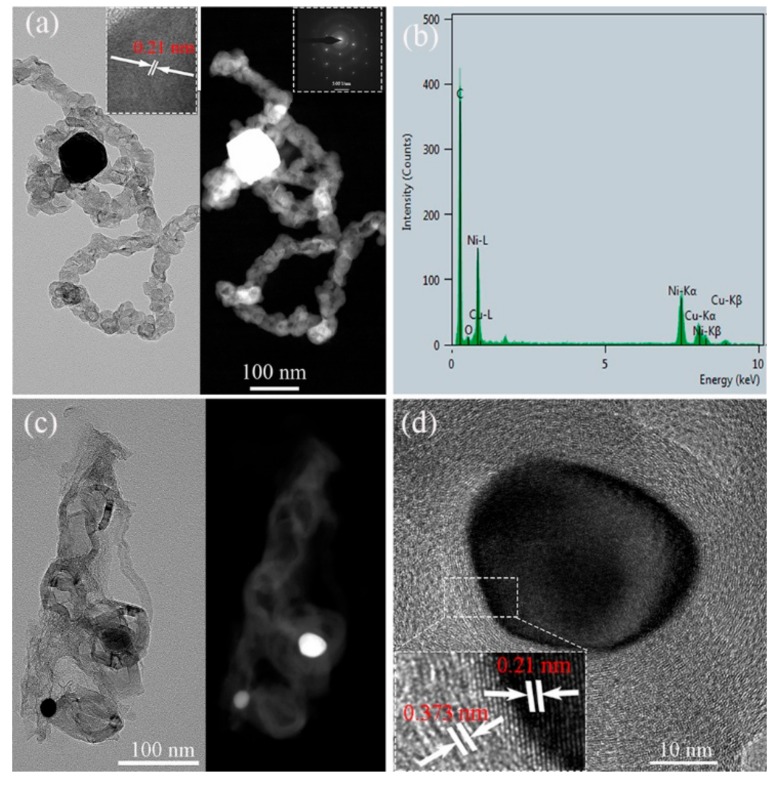
TEM bright-/dark-field images and SAED (inset) of samples: (**a**) Hollow CNTs and (**b**) EDS pattern, (**c**) bamboo-like CNTs, and (**d**) HRTEM image.

The morphologies of the products with and without nickel nitrate (Figure 7, Figure 9 and Figure 10) revealed that nickel nitrate played a key role in the formation of CNTs from a catalytic pyrolysis of phenolic resin. Therefore, the position and structure of Ni in the two CNT types should be studied. The TEM bright/dark-field and HRTEM images are shown in Figure 12. Along the bamboo-like chain CNTs (Figure 12a), some bright Ni particles (dark-field images) were concentrated inside and on the CNTs (EDS in Figure 12b). Furthermore, the SAED proves that it was a single-crystal Ni with a lattice spacing *d* of 0.21 nm, which corresponds to the (111) plane of Ni (inset in Figure 12a) [39]. However, hollow CNTs with only superficial Ni were also observed (Figure 12c). Furthermore, the Ni (*d* of 0.21 nm) was covered by carbon (*d* of 0.373 nm) (Figure 12d).

**Figure 13:**
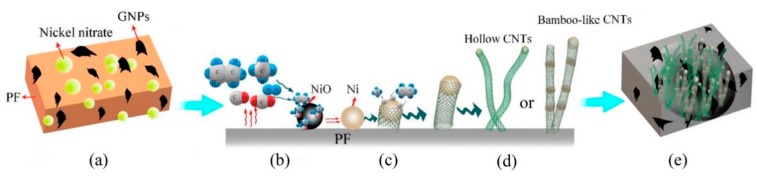
Schematic diagram of growth process of CNTs from catalytic pyrolysis of phenolic resin with Ni (**a**) The exfoliated GNPs/PF sample with 1 wt.% Ni(NO_3_)_2_·6H_2_O, (**b**) the formation of metallic Ni, reduced from the NiO by reductive atmosphere from the pyrolysis of the PF, (**c**) the precipitation of CNTs across the Ni bottom, (**d**) the growth of hollow or bamboo-like CNTs, (**e**) the formed CNTs were uniformly distributed in the pyrolyzed sample.

Generally, there are two well-established growth mechanisms based on the catalyst location in the CNTs: Base and tip growth mechanisms [40,41,42]. The state of a catalyst is determined by its melting point and has great effects on the growth of CNTs. Based on the catalyst state, vapor–solid (V–S) and vapor–liquid–solid (V-L-S) mechanisms govern the growth of CNTs prepared through a chemical vapor deposition (CVD). Therefore, the growth mechanism depends on the preparation method and the environmental conditions. In this study, the melting point of the Ni nanoparticles as the catalyst was calculated based on the following equation:

(1) $$\Delta T=\frac{2\gamma \mathrm{MT}}{r\rho Q}$$

where ∆*T* is the reduced temperature at the melting point, *γ* is the surface tension, *M* is the relative molecular mass, *T* is the melting point, *r* is the diameter of the particles, *ρ* is the relative density, and *Q* is the Moore latent heat of fusion [43,44]. For Ni as the catalyst, *γ*, *M*, *T*, *ρ*, and *Q* were 1.796 J·m^−2^, 58.7 g·mol^−1^, 1726 K, 8.9 g cm^−3^, and 17.47 kJ mol^−1^, respectively. The diameters *r* of the Ni particles were 10–100 nm (Figure 10a,c). According to the calculation, the reduced temperatures ∆*T* of the melting point were 23.4 K (*r* = 100 nm)–234.1 K (*r* = 10 nm). Thus, the melting points of the Ni nanoparticles occurred at 1491.9 K (*r* = 10 nm)–1702.6 K (*r* = 100 nm), which were much higher than the treatment temperature (1273 K) adopted in this study [45]. Moreover, the eutectic point of the Ni–C binary system was 1599 K based on the equilibrium phase diagram, which was also higher than the treatment temperature. Evidently, the nanoscale Ni grains as the catalyst remained in a solid state in this study. Thus, the growth process of the CNTs via the catalytic pyrolysis of PF should be dominated by the V–S growth and tip growth mechanisms (Figure 13).

According to the TG/DTA results, the growth process of the CNTs through the Ni(NO_3_)_2_·6H_2_O catalytic pyrolysis of PF can be divided into three main stages based on the increasing temperature. First, the nickel nitrate was decomposed to form NiO (Equation (2) and Figure 13a,b). Simultaneously, the PF pyrolysis released H_2_, CO, CH_4_, C_2_H_6_, and CO_2_ gases (Equation (3) and Figure 13b). The resulting NiO was reduced to metallic Ni by the reductive atmosphere (i.e., H_2_, CO, CH_4_, and C_2_H_6_) (Equation (4) and Figure 13b). For the growth of hollow CNTs, at the early stage, the volatile components of hydrocarbon were adsorbed at the top of the surface of the catalyst particles and then decomposed into carbon atoms at a certain temperature range. Soon afterward, the formed carbon atoms diffused down through the metallic catalyst to the catalyst/substrate interface, which thereby formed some proto-graphene. Finally, the CNTs were precipitated across the Ni bottom (Equation (5) and Figure 13c) [46,47]. This nucleation step led to the elongation of the particles. Thus, Ni was lifted off from the PF substrate (Figure 13c). As long as the Ni activity enabled the carbon diffusion, the CNTs continued to expand. However, as the diffusion carbon atoms covered the Ni entirely, the Ni catalysis ceased, and the CNTs growth stopped (Figure 13d). Regarding the growth mechanism of the bamboo-like CNTs, the only difference was that the bamboo knots within the CNTs preferentially nucleated on the multistep Ni–carbon edges. Furthermore, the growth of a complete inner few-graphene layer prior to the Ni particle contraction to restore cohesive forces resulted in bamboo-like CNT knots [48].

(2) $$\mathrm{Ni}{\left({\mathrm{NO}}_{3}\right)}_{2}\cdot 6H_{2}O\to \mathrm{NiO}+{\mathrm{NO}}_{2}+O_{2}+H_{2}O$$

(3) $$C_{7}H_{6}O_{2}\to H_{2}+{\mathrm{CH}}_{4}+C_{2}H_{6}+\mathrm{CO}+{\mathrm{CO}}_{2}$$

(4) $$\mathrm{NiO}\overset{\mathrm{CO}/H_{2}/{\mathrm{CH}}_{4}/C_{2}H_{6}\;}{\to }\mathrm{Ni}$$

(5) $$C_{x}H_{x}\overset{\mathrm{Ni}}{\to }\mathrm{CNTs}$$

## 4. Conclusions

In this study, the structure of FG in PF exfoliated with the TRM technology and the catalytic pyrolysis of PF with Ni(NO_3_)_2_·6H_2_O at 1000 °C were investigated. The following conclusions can be drawn:

(1) The TRM method enabled the simple exfoliation of FG into micro/nano graphite platelets (GNPs) in high-viscosity PF. With an increasing exfoliation time, the sizes and thicknesses of the products decreased gradually. The micro/nano GNPs exhibited sizes of 10–30 μm and thicknesses of 30–50 nm when exfoliated 16 times.
(2) After the GNPs/PF were pyrolyzed in an argon atmosphere at 1000 °C for 3 h, the GNPs were uniformly dispersed and closely embedded in the GC originating from the PF pyrolysis.
(3) The Ni(NO_3_)_2_·6H_2_O played a key role in the formation and growth of CNTs converted from the catalytic pyrolysis of partial PF. The resultant multidimensional carbon nanostructures with GNPs/GC/CNTs were obtained, owing to the shear force of the TRM process, pyrolysis, and catalytic action of nitrates. These results provide a novel method for the production of inexpensive carbon precursors that can be applied in LCCRs.
